# The African Pitocin - a midwife’s dilemma: the perception of women on the use of herbs in pregnancy and labour in Zimbabwe, Gweru

**DOI:** 10.11604/pamj.2016.25.9.7876

**Published:** 2016-09-19

**Authors:** Tsitsi Panganai, Precious Shumba

**Affiliations:** 1 Zimbabwe Open University, Nursing Sciences, Zimbabwe; 2 Bsc Nursing, RGN SCM Claybank Hospital, Zimbabwe

**Keywords:** Labour, herbs, pregnancy, safety, midwife

## Abstract

**Introduction:**

The use of natural health products is gradually increasing all over the world with up to 50% of the general population having tried at least one herbal product. This becomes a dilemma to the midwife who has limited or no knowledge on their effects in pregnancy, hence the need to explore the perceptions of women on the use herbs in pregnancy and labour.

**Methods:**

The research, which was a case study of a Claybank Private Hospital in Gweru, Zimbabwe, adopted a qualitative approach with a triangulation of data from interviews, observations and analysis of maternal records. A sample of 20 women, admitted to using herbs, was purposively selected from the labour and post natal wards.

**Results:**

A variety of substances, but mainly the elephant's dung, was used. The family, (mother) prescribed the herbs. The women did not have knowledge on how the substances work but believed in them, as they have stood the test of time.

**Conclusion:**

The African women in Zimbabwe cannot be stopped from taking herbs as it is engraved in their culture and have absolute faith in them. Whilst the herbs are assumed by the women to be effective, their safety is questionable, especially in women with underlying obstetric complications. It is therefore recommended to scientifically explore the safety and effectiveness of the most commonly used herbs if pregnancy is to be safe. Whilst the women can not be stopped from taking these herbs, it is important to build a trusting relationship between the midwife and the mother so that communication about the use of herbs can be done freely without fear or judgement.

## Introduction

The uses of alternative therapy which include herbs have taken the world by storm and many women are now taking herbs during pregnancy and in labour. All over the world, up to 50% of the general population has tried at least one herbal product. It is estimated that 85% of the women depend on traditional healthcare systems for their beneficial effects during pregnancy, birth and postpartum care [1, 2]. However, its use [3] may lead to foetal distress and other obstetric complications. Zimbabwean women use herbal medication during pregnancy, labour and after birth and this is a dilemma for a midwife in Zimbabwe as one is never sure of what the women has taken or its effects to the baby and mother. The question therefore is why do women take these herbs? During pregnancy and childbirth, traditional birth attendances rely on the use of certain herbs for their beneficial effects to tone the uterus muscle, induce labour, and remove a retained placenta and management of post-partum bleeding [4]. However [5], emphasizes that the safety of herbal drugs is particularly important in pregnant women and children. In support of this [3], found that 55% of the 229 women interviewed gave a positive history of herbal ingestion and 55.6% of them had grade II-III foetal distress compared to 15% in the control group. In addition, 38.5% of the study group delivered by caesarean section as opposed to 22% of the control group. The safety of these herbs is therefore questionable but the women continuously use them. Could it be that they have some knowledge that is not open to everyone?

On the other hand, [6] claim that women die during pregnancy and childbirth annually and many of these women would not have receive the necessary maternal care due to socio-economic factors but they have access to medicinal plants, which potentially could save them [7]. Whilst exposure to herbal products is frequent in women [4], there is insufficient data available to justify herbal use during pregnancy. Never the less [8], documentation and biological validation of traditionally-used herbal remedies are ideal starting points for biological target-oriented drug discovery efforts and their pharmacological characterization may eventually lead to the development of novel uterotonic drugs. However these herbs need to be known first. Herbal medicines are preparations derived from naturally occurring plants with medicinal or preventive properties [9]. Herbal drugs have the potential to elicit the same types of adverse reactions as synthetic drugs, since they consist of the whole extracts or, more of defined parts of plants (roots, rhizomes, leaves, and flowering heads) that contain numerous active molecules [10]. The safety of herbal drugs becomes particularly important in patients such as pregnant women and children, who are more vulnerable to the effects of drugs due to their physiological characteristics [11]. Pregnancy care providers should be aware of the common herbal supplements used by women and of the evidence regarding potential benefits or harm [8]. In spite of the fact that side effects and teratogenic potentials of most herbal products are poorly understood, indiscriminate use of herbal remedies in different forms is very rampant.

The use of herbal drugs is widespread in countries like India and China, and in large parts of Asia, South America and Africa. The actual incidence of this use is unknown, even if it has been reported by some authors as varying between 7 and 45% depending on geographical area [3, 12]. Herbal usage has also spread to the western countries where it is referred to as alternative therapy. Although it is recommended to avoid herbal products during pregnancy, many women use herbal products during pregnancy. There are complex reasons for the preference of herbal medicine and some of them are associated with cultural and personal beliefs as well as philosophical views toward life and health [10] as well as comparison of experiences between conventional healthcare professionals and complementary medicine practitioners by patients [13]. A Canadian study on pregnant women found that the women considered herbs to be safer than pharmaceuticals because they were “milder”, “more natural”, “simpler”, “more familiar” or “caused fewer side effects” [14]. An Italian survey showed that herbal use was increasing and that herbal products are considered to be safer than conventional drugs because they are natural [15]. But is it so? It has been noted that pregnant women recognize the potential risks of drug consumption, but do not realize that herbal products, if taken incorrectly, could also be toxic. Knowledge of potential side effects of many herbal medicines in pregnancy is limited [3, 16]. How do the women get these herbs?

Herbal drug usage is mostly recommended by family (87.3%) and rarely by primary maternity care providers (7.6%). Family members who mostly recommended the use of herbal may not have sufficient knowledge to advise pregnant women about the use of herbal drugs [17]. Birth attendances [18] are also a source of herbal information for pregnant mothers. Since the family is core to the use of herbs, it can be assumed that pregnant women are reluctant to discuss herbal usage with Health care personnel. In a study by [19], physicians were questioned about herbal drugs and 80.7% rated their knowledge as poor. Similar studies done in England and the Netherlands produced similar results [20]. In the same vein, a study by [21] found that physicians are less likely to recommend herbal products to pregnant and breastfeeding women than alternative practitioners. As a result women do not discuss their use of herbs with the doctor or midwife. An Australian study found that more than half of the pregnant women did not report their use of herbs a health worker prescribing conventional medicines [22], yet is expected to be attended by that person. Such secrecy compromised the quality of nursing care rendered to and makes nursing these women a dilemma. Whilst it maybe a dilemma to the midwife, what are the perceptions of the women who take herbs. The objectives of the study were: to analyse the use of herbs in labour; to identify the types of herbs used during labour; to find out the source of the information on herbal usage; to evaluate the knowledge of the women on the herbs they take.

## Methods

**Research design:** the research was conducted as a case study of women attending maternal services at Claybank Private Hospital in Gweru, Zimbabwe. A case study was chosen as it allows an investigation of a phenomenon within its real life context without manipulation of behaviour. The study is done over time and in this case over six months Claybank Private Hospital was chosen because one of the researchers works there so it was easy to collect data over a period of time. It adopted a qualitative approach as it allows investigation of a phenomena, understanding of the human behaviour and reasons that govern such behaviour. The qualitative approach enables the study of people in their natural setting.

**Study setting:** the study was carried out at Claybank Private Hospital in Gweru urban, Zimbabwe. This private hospital has a very busy maternity department catering for the middle income group of women from all over the midlands province. It was also easier to collect data from the clients as the one of the authors was working at this hospital at the time. It was because of these reasons that this hospital was chosen to be the study site.

**Population and sampling:** The study population were women in labour who had admitted to taking herbs during pregnancy and labour. The study population also included post natal mothers attending post natal clinic, and likewise, had admitted to take herbs. The purposive sampling method was used to select a sample of 20 women who admitted to have taken some herbs during pregnancy and delivery. However it took a lot of persuasions and reassurances for the women to admit to taking herbs. This method of sampling was used as the researchers wanted to get first hand information from the women who had actually used the herbs.

**Instruments and data collection:** Data was collected using interviews, observational methods and analysis of maternal records. Interviews were chosen as they allowed probing of interesting responses and observation of body language. They also allowed the researchers to explain the purpose of the study so as to obtain verbal consent. The author who was working in the maternity ward asked all the mothers she came across about the use of herbs and only to those who verbalised the use of herbs were interviewed. Data obtained from the interviews was triangulated with observation on the progress and outcome of labour and analysis of maternal records of the women who had taken herbs in an effort to improve validity of the findings. The data collected from interviews, document analysis and observations was analysed thematically according to the objectives so as to bring out the perceptions of women on the use of herbs. Quotes were used to support the emerging themes.

**Ethical issues:** Permission to carry out the study was sought from the hospital authorities and the women were reassured of anonymity and confidentiality once their consent was given. Permission to carry out the research was sought at Claybank Hospital and the medical research council of Zimbabwe.

## Results

A number of themes emerged from the study and the data was analysed following the themes.

### Use of herbs in labour

The study findings revealed that the use of herbs in labour and during pregnancy is engraved in the culture of the participants. Ninety (90%) of the participants said that they use herbs as it is their culture to prepare for the baby. They argued that their mothers and those before them used these herbs and things worked for them so why would they not also use the same old herbs. Some of the sentiments raised by the women were: *“A mother would be labelled a fool if she does not give her daughter herbs to use during pregnancy and in labour”. “These herbs have been tried and tested so we use them”. “The herbs we use in our family were carried over from previous generations”.*

However, there were some (10%) who took the herbs against their will. They were literally forced to take the drugs or face the consequences of loosing a baby or dying during labour. *“I took the herbs because I was scared of being blamed if something wrong was to happen during labour,”* said one of the women who took the herbs against her personal belief. All (100%) of the women who took the herbs never discussed it with the health workers. Taking of these herbs was regarded as top secret amongst the family members. The participants claimed that the nurses did not understand the use of the herbs, neither did they like it when labour does not progress the way they want. The following sentiments were verbalised by most of the women: *“The nurses will scold you if you tell that that you took nyeluka (herbs that speed up labour)”. “The nurse who delivered me suspected that I had taken nyeluka and was very angry but I denied it”. “When my labour progress very fast the nurse got angry but I pretended to be innocent”.*

Labour records of ten (10) women who had admitted to taking herbs for labour were analysed. Of these 10 women, 7 (70%) had caesarean section (C/S) done for fetal distress, and one of them had uterine rapture. Three (30%) had normal delivery but one had post partum haemorrhage due to uterine atony. One of the researchers, a midwife working in Labour Ward, observed that the contractions of the women who had agreed to taking *nyeluka*, were very strong in relation to the cervical dilatation. There were 5- 6 contractions in 10 minutes when the cervix was only 4 -6 cm dilated on three of the observed clients. The labour of one of the clients was progressing well until after visiting time when the contractions became very strong 6 in 10 minutes whist the cervix was 6 cm dilated. The foetus was distressed and she ended up going for C/S. She admitted that her mother had given her some herbs to speed up labour during the visiting time. Vaginal examination done by the researcher during labour revealed that the membranes were adherent to the presenting part on all the five women who were delivered by the researcher. Four of the clients nursed by the researcher had foetal distress. The foetal distress could be attributed to the strong contractions.

### Types of herbs used during labour

The participants mentioned a variety of herbal preparations that they used in pregnancy and labour. However some of the participants did not even know what they took but took it because they had faith in the person giving them the preparation. Table 1 shows some of the herbs, preparation and purpose mentioned by the participants. The most popular preparation used in labour was the elephant's dung which was mentioned by 80% of the women. The other preparations were mentioned by 10% and the remaining 10% did not know what they were given.

**Table 1:** Herbal preparations used by african women in pregnancy and labour

HERB	PREPARION	PURPOSE
Elephant’s dung	Soak it in water and drink at the onset of labour	Quickens labour
Leaves of Mutohwe(Sn ot apple)	Crush and soak in water	Induces labour
Leaves of Mufute	Crush and soak in water	Augments labour
Chifumuro (Dicoma anomala Sonda)	Crush and soak in water /chew	Overcomes labour complications related to witchcraft
Ruredzo (dicerocaryum zanguebarium)	Soak in water, lubricate a fist and push it in the vagina	Widen the vagina and makes birthing easy
Mutsvengo/Nhanzva(Pouzolzia mixta Solms)	Roots extract instilled into the vagina	To dilate birth canal
Sweet potato leaves	Boil the leaves and drink the fluid from 36/40 daily until onset of labour	Quicken labour
Rukato(Asparagus Africanus)		Aid in child birth

### Source of the information on herbal usage

The study identified the woman's family (90%), as the main source of information on herbal use. Even if the family, (mother, grandmother and aunties) did not know the herbs, they were the ones who would seek information from the traditional healers, friends or neighbours. *“My mother did not know the herbs but was given by her friend,”* said one participant.

### Knowledge of the women on the effects herbs they take

The participants were not very sure of the effects of the herbs they took. They simply took them because they have faith in their elders. However, from the following sentiments, the women believed that the herbs work. *“These herbs have been tested over time”. “My mother told me that nothing wrong will happen after taking “chifumuro” and for sure nothing happened”. “After taking the elephant's dung, my labour progressed so fast that the nurses asked me if I had taken “nyeluka” ”. “My labour progressed very fast from 3cm to fully dilated within 2 hours”.*

Nevertheless, the herbs did not work for some of the women. One of the participants said that she was forced to take *nyeluka* but had caesarean section (C/S) for cephalo-pelvic disproportion (CPD). On the other hand, one participant who took *chifumuro* believed it worked because she was scheduled for C/S but delivered normally. Another woman took *mufute* and the labour progressed very fast but had C/S because the baby was not descending and the child died after 24hours. *“When I told them that I want to push they said the baby was tired and that I should have an urgent C/S”*

The study findings also showed that previous experience can make one take these herbal preparation. Some women took the herbs after experiencing a difficult delivery with their previous pregnancies, and labour progressed well. *“I had C/S for my first baby but after taking chifumuro during pregnancy and nyeluka during labour, I had a very easy normal delivery”. “I did not take anything with my first baby and I spent 3 days in labour so I took the elephant’s dung this time and only spent 6 hours in labour”. However, the study brought out some element of myths and misconceptions surrounding the C/S “I was bewitched that is why I had a C/S”.*These sentiments show that these women have faith in the herbs than the conventional healthcare which according to them failed.

## Discussion

### Use of herbs in labour

The participants had absolute trust in the herbs they use and this was a result of their culture. These cultural beliefs are passed from generation to generation so herbs will always be a part of these women's lives and care. A mother takes it as her responsibility to ensure that she prepares her daughter for delivery. The findings also concurred with [10], who found that the use of herbal medicines is associated with cultural and personal beliefs as well as philosophical views on life and health. However, the issue of culture is so strong that even those women (10%) whose personal beliefs and philosophical views did not allow them to use herbs took them because their culture expected them to do so. When it came to labour and pregnancy, cultural beliefs override personal beliefs. It was evident from the study that there is no therapeutic communication between the mother and the midwife in terms of the use of herbal preparation. The midwife was left in the dark and had to guess or assume that herbs were taken when the progresses of labour deviates from the normal. The findings are similar to those of [21, 22], who found that pregnant women did not report their use of herbs a health worker prescribing conventional medicines. It is because of this lack of trust between the client and the midwife that midwifery becomes a dilemma as the nurse is never sure of what has been taken and what is coming. The element of trust becomes an issue since the findings indicated that nyeluka could have adverse effects on the fetus and the uterine muscles. The findings support [23] who identified children as particularly at risk from the use of herbal medicines as they are generally untested. In addition [3], also found that 55.6% of women who had taken herbs had grade II-III foetal distress compared to 15 % in the control group. In addition, 38.5% of the study group delivered by caesarean section as opposed to 22% of the control group. Whilst the women had absolute trust in their herbal preparation, they could be harmful so there should be open communication with the midwife who should be alert to early signs of foetal distress and intervene accordingly. Through open communication, the herbs taken can be documented and this could be the basis of studying their effects. However more research in needed to study the effects of herbs in labour so that the mothers are advised accordingly.

### Sources of information

The maternal family of the woman has a mandate to prepare their daughter for delivery hence is responsible for looking for the herbs. According to [17], herbal drug usage is mostly recommended by family (87.3%) and rarely by primary maternity care providers (7.6 %). In the participants' culture, a woman delivers her first child at her own family's home making it easy for the family to supply her with the herbs. At the same time the family's cultural beliefs related to pregnancy, delivery and child rearing are passed on to the young mother who will in turn pass it to her daughter/s thus perpetuating the beliefs. It can be assumed that, traditional birth attendances, by virtue of them being the “midwife”, are considered to be experienced hence are consulted by the family. A study by [18], also found birth attendances to be the source of herbal information for pregnant mothers. These TBAs are elderly women of the community who commands respect so it is culturally recommended to listen to the elders as they know best. Because of this, they become herbal consultants in the community. However, these herbs do not have a dose so it becomes very easy to overdose a woman who will end up with complications. They need to be tested to prove their efficacy.

### Knowledge of the effects of herbs

From the findings, the herbs maybe beneficial to others but it could be risky if there are some underlying complications. When the women took the herbs, they did not discuss with their health workers to rule out underlying problems like CPD which could be fatal in the herbs induced precipitate labour. Such women ended up going for C/S which is surrounded by myths and misconceptions as the women have blind faith in their herbs. From the findings, it was apparent that the women did not attribute labour complications to herbs. Such behaviour is an indication of lack of knowledge on the effects of herbs or indications for C/S. One of the women who had C/S actually attribute it to some witchcraft or fate. Some of them even blamed the midwives for their misfortunes saying, “*the nurse did not deliver the baby in time. They wanted to fix me and sent me for C/S.*” Unless such myths and misconceptions are removed from these women, the dilemma that the midwives are facing will always be there. These findings were similar to [18], who claim that family members who mostly recommended the use of herbal may not have sufficient knowledge to advise pregnant women about the use of herbal drugs. However, a difficult labour can make one seek alternative therapy even if not sure of how it works. What this means is that those women who had a difficult labour are at risk of using herbs so that labour becomes easy. The findings concur with [13] who also found that comparison of experiences between conventional healthcare professionals and complementary medicine practitioners by patients makes them decide otherwise.

### Strengths and weakness of the study

The study managed to prove that the women can discuss the issue of herbs. It is a just a matter of patients and attitudes of the midwife. If the midwives take their time and discuss these issues without being judgemental, communication could be opened since the participants confided to the authors who are midwives with one of them working at the institution. The study also managed to unravel a number of the herbs which are dubbed the “African Pitocin”. By identifying these herbs, they can be scientifically analysed so that their effects are known. Once their effects are known, they can either be used officially or scraped completely. The midwives will be able to give scientifically proven information about the herbs. However, the sample was too small to generalise the information. More research need to be done in various set ups and not just one private hospital.

### Implications of the study

As the use of herbal medicine has become more prevalent, health care personnel need to be aware of potentially beneficial as well as harmful effects related to their use. Just like with any over-the-counter or prescription medicine. If safe motherhood is to be achieved the healthcare provider should be kept informed of the use of herbal therapy, and preferably the woman should discuss the use of herbs before she actually starts using them. Health care personnel should ask pregnant women if they take any herbs, or if they plan to. This should then be documented and the woman be closely monitored. The health care personnel should also familiarize themselves with how specific herbs are used, because the same key concepts underlying the administration of medications apply to herbal medicines as well, i.e. right medication, right route, right dose, and right time. The midwives need to be aware of the common herbal preparations used by women, and of the evidence regarding potential benefits or harm. Discussing the use of herbs in an open and nonjudgmental way will go a long way toward helping the patient and provider to communicate effectively about this topic. It is essential that health care personnel teach their patients about possible interactions between herbs and prescription or over-the-counter medications. It is, however, important that midwives do not prescribe any treatments, medications or herbal supplements where they are unaware of the evidence supporting their safe use. As a result, the herbs mentioned by the women need to be studies to evaluate their usefulness in obstetrics and their adverse effects because if they are useful they solve the financial difficulties encountered by women in accessing medical care. In addition, these drugs need to be recorded before the information is lost due to lack of knowledge by the young generation

### Unanswered questions

From the study there were questions which were not answered and some of them are: what is the pharmacokinetics of the herbs taken in pregnancy and in labour? What are the perceptions of the midwives who nurse these clients? How can communication be improved? These questions are the basis for further research on use of herbs in pregnancy and labour.

## Conclusion

From the findings, it is difficult to say that the herbs are effective and safe as the women would like to claim. Some women had precipitated labour after taking *nyeluka* whilst others experienced complications. Yes there may be perceived or actual effectiveness in terms of shortening labour, but are not safe especially if they are taken with women who have CPD. It is therefore crucial to scientifically test these drugs if pregnancy is to be safe. Since the women can not be stopped from taking these herbs as it is engraved in their culture and some have absolute faith in them, communication can be improved. What is important is to build a trusting relationship between the midwife and the mother so that communication about the herbs can be done freely without fear or judgement. Open communication would be beneficial to both sides as the midwife would be alert to any deviation from normal labour and action would be implemented in time thus resulting in safe motherhood.

### What is known about this topic

- Herbs can have adverse effects on the baby;
- The use of herbs is part of the African culture which is practiced to ease the process of labour.

### What this study adds

- Herbs can alter the outcome of labour in both positive and negative ways;
- Herbs are trusted more than the conventional medicines even though the therapeutic effects are not known;
- Elephant's dung is a very popular preparation used to speed up labour.
